# Adults’ dental treatment in 2001–2013 in Finnish public dental service

**DOI:** 10.1186/s12903-020-01091-w

**Published:** 2020-04-21

**Authors:** J. Linden, E. Widström, J. Sinkkonen

**Affiliations:** 1 Public Dental Service Lohja, Lohja, Finland; 2 Institute of Clinical Dentistry, Arctic University of Norway, Tromso, Norway; 3 National Institute for Health and Welfare (THL), Helsinki, Finland; 4 Finland Reaktor ltd, Helsink, Finland

**Keywords:** Registers, Oral health, Treatment needs, Public dental service, Treatment measures

## Abstract

**Background:**

All adults over 17 years of age have access to the Public Dental Service after the Finnish Dental Care Reform in 2001–2002. This study aimed to survey the treatment needs and treatment measures provided for adult patients and changes in these during the period 2001–2013.

**Methods:**

Sing each person’s unique identifier, demographic data on dental visits during the period 2001–2013 were collected from municipal databases in five PDS-units covering 320,000 inhabitants. The numbers of visitors, those in need of basic periodontal or caries treatment (CPI > 2 and D + d > 0) were calculated for three age groups. Treatment provided was also calculated in 13 treatment categories. Trend analyses were performed to study changes during the study period.

**Results:**

Restorative treatments (968,772; 23.6%), examinations (658,394; 16.1%), radiographs taken (529,875; 12.9%) anaesthesia used (521,169; 12.7%) and emergency treatments (348,229; 8.5%) made up 73.8% of all treatment measures during the entire study period. Periodontal treatment (7.8%) and caries prevention (3.9%) made up a small part of the care provided and prosthetics and treatment of TMJ disorders were extremely uncommon (fewer than 1%). Treatments related to caries (restorative treatment, examinations, endodontics, emergencies, anaesthesia and radiographs) made up 60.4% of the dental personnel’s treatment time. During the study period, statistically significant increasing trends were found for radiographs (*p* < 0.001***), anaesthesia (*p* = 0.003**) and total number of treatments (*p* = 0.009**). There was a slight decreasing trend in treatment need among the youngest adults (18–39 years; *p* = 0.033*).

**Conclusion:**

Compared with the results of national epidemiological studies, insufficient periodontal treatment is provided and prosthetic treatment is almost totally neglected in the PDS. Rather, adults’ dental treatment concentrates on treatment of caries. The unmet needs may be due to tradition, inadequate treatment processes or a lack of resources or failed salary incentives.

## Background

In Finland, adults’ oral health has been monitored by three nationally representative clinical epidemiological studies in 1980, 2000 and 2011 [1–3]. These studies have shown that adults in general have poor oral health: they have lost many teeth and edentulousness is still common, especially among the elderly. Caries prevalence has decreased slightly [1–3] but the periodontal conditions have not improved during recent decades [1–4]. Home care habits are not good [3]. In the neighbouring countries, Sweden and Denmark progress has been much better [5, 6]. In Germany, adults have better oral health [7].

Since the early 1970s, the Public Dental Service (PDS) has catered for most children and adolescents younger than 18 years. It offered examinations, prevention and all necessary care free of charge [8]. Since the 1980s, adults were successively given access to the subsidized dental services in the PDS, starting with the 18–25-year-olds youngest age groups. Older adults were assumed to visit private dentists or clinical dental technicians (denturists) and pay for their treatments out-of-pocket [9].

The Dental Care Reform in 2001–2002 abolished all age restrictions and persons older than 46 years (born before 1956) were allowed to book appointments in the PDS [9]. After the onset of the Reform from 2001 to 2007, adults’ use of oral health services increased, perceived need for oral health care decreased and socioeconomic inequity in use of care decreased. However, socioeconomic inequalities in reporting the need for emergency care increased [10]. In the PDS, a third (36.4%) of all treatment measures were still provided for children and adolescents in 2009 and waiting lists for adults were long [11]. About half (48.5%) of the working aged (18–64-year-olds) who visited a dentist in 2009 had used private services and the other half (51.5%) public services. Of the elderly (65+ years), 56.9% had visited private dentists and 43.1% public dentists [12].

There are few studies on dental treatment provided in general [6, 13, 14] and especially in a longitudinal perspective. Overall, developing outcome measures for oral health care and using them for evaluation and steering purposes is still just beginning [15].

The aim of this study was to survey treatment needs and treatment measures provided for adults over 17 years old in the Public Dental Service and changes in them during a 13-year period from 2001 to 2013.

## Methods

As described in our previous article [8] we asked five PDS units in southern Finland, where the same specific electronic patient registration system [16] was in use, were asked to participate in the study. Ethical approval was provided by the National Institute for Health and Welfare (THL 1697284289204448) and permission to use the local data was granted by the directors of health services in each PDS unit. The total number of adult inhabitants (> 17 years) in the participating PDS units’ catchment areas was in 2001, 240,584 and in 2013, 262,703 persons [17].

Data on all the adults (> 17 years) who had visited the five PDS units during 2001–2013 were collected retrospectively from each municipal database. For each year, the numbers of all patients who had visited a dentist and all treatment measures provided by any professional category (dentists, dental hygienists and dental nurses) were extracted from the databases [8]. Data on need for basic periodontal or caries treatment (CPI > 2, D + d > 0) [18] were also collected.

The patients were grouped into three age categories (18–39 years, 40–64 years and 65+ years). The items of treatment provided were classified into 13 main treatment areas: clinical examinations including complementary examinations (laboratory tests etc.), preventive care (instruction of oral hygiene, dietary advice, fluoride varnish etc.), periodontics (scaling etc.), restorative care (permanent and temporary fillings, crowns of filling material), endodontics, treatment of temporomandibular disorders (TMD), orthodontics, prosthetics (crowns, bridges, removable dentures etc.), anaesthesia (local anaesthesia, sedatives, nitrous oxide), emergency treatment, radiology, oral surgery and other treatment (removal of sutures, local medications, certificates etc) [8].

To control the possible effect of some treatment measures being short and others time consuming, all treatment measures collected were converted into treatment time (minutes, hours) using the average durations of the treatment measures as observed in a recent Finnish study [19].

The R 3.3 environment for statistical computing was used for descriptive and inferential analyses. Annual numbers of patients, the numbers of the examined, those in need of treatment and sum of treatment categories as well as their proportions of total are presented [8]. Numbers of treatment measures per 1000 patients combined by age group are also presented.

To discover underlying trends, we modeled volumes of patients in treatment categories, total treatment need, agreement of treatment needs and the volume of preventive treatment as functions of year. After logarithmic transformation of volumes, our linear models assume constant percentage change over time, with deviations from the mean normal on the log-scale and with explicitly autocorrelated residuals [20]. Fits to data were adequate except for very low-volume categories of TMJ disorders and prosthetics. Significances are reported at the level *p* < 0.05 [8].

## Results

From the first study year (2001) to the last (2013), the number of adults having visited the PDS increased by 81.5% from 37,377 to 67,834. The number of patients in the youngest age group (18–39-year-olds) increased only by 6.5% from 25,463 to 27,113. The age group 39–64-years-old increased by 183.5% from 9760 to 27,666 and the oldest group (65+ years) increased five-fold from 2154 to 13,055 (Table 1). The total number of adults treated during the 13-year study period was 203,619 (Table 1). This means that about 77.5% of the adult population had visited the PDS on one or more occasions during 2001–2013.

**Table 1:** **Table 1** Numbers and distribution (%) of adults (> 17 years), total and by age group (18–39 years, 40–64 years and 65+ years) treated in the five PDS units, and numbers of treatment measures by treatment category provided for them by year and totally during 2001–2013 as well as the change between 2001 and 2013 (%)

Year	2001	2002	2003	2004	2005	2006	2007	2008	2009	2010	2011	2012	2013	Sum (%)	Change from 2001 to 2013
Patients, all and number in the category														Total number of different individuals in age category during the 13 years.	
All (N)	37,377	37,862	38,566	39,313	43,414	48,689	46,331	47,540	55,074	57,021	58,612	64,816	67,834	203,619	81,5
18-39 year olds(%)	25,463 (68%)	23,138 (61%)	21,130 (55%)	19,836 (50%)	20,559 (47%)	21,979 (45%)	19,925 (43%)	20,160 (42%)	22,374 (41%)	23,020 (40%)	23,038 (39%)	25,745 (40%)	27,113 (40%)	119,549 (58.7%)	6,5
40-64 year olds(%)	9,760 (26%)	12,291 (32%)	14,054 (36%)	15,290 (39%)	17,554 (40%)	19,778 (41%)	19,387 (42%)	20,117 (42%)	23,719 (43%)	24,448 (43%)	25,055 (43%)	26,929 (42%)	27,666 (41%)	76,399 (37.5%)	183,5
65+ year olds(%)	2,154 (6%)	2,433 (6%)	3,382 (9%)	4,187 (11%)	5,301 (12%)	6,932 (14%)	7,019 (15%)	7,263 (15%)	8,981(%)16	9,553 (17%)	10,519 (18%)	12,142 (18%)	13,055 (19%)	32,134 (15.8%)	506,1
Examined (per cent of patients)															
18-39 year olds(%)	17,340 (68%)	14,600 (63%)	12,032 (57%)	10,146 (51%)	11,614 (56%)	11,043 (50%)	8,005 (40%)	9,162 (45%)	9,403 (42%)	10,141 (44%)	9,933 (43%)	11,597 (45%)	11,131 (41%)	85,997 (71.9%)	-35,8
40-64 year olds(%)	5,479 (58%)	6,634 (54%)	7,139 (52%)	6,850 (45%)	8,886 (51%)	9,141 (46%)	7,114 (37%)	8,451 (42%)	9,271 (39%)	9,580 (39%)	9,821 (39%)	11,064 (41%)	10,951 (39%)	51,570 (67.5%)	99,9
65+ year olds(%)	1,071 (50%)	1,184 (49%)	1,829 (54%)	1,807 (43%)	2,300 (43%)	2,648 (38%)	2,251 (32%)	2,810 (39%)	3,517 (39%)	3,932 (41%)	4,508 (42%)	5,746 (47%)	6,213 (48%)	20,278 (63.1%)	480,1
In need of basic treatment (per cent of the examined)															
18-39 year olds(%)	13,070 (75%)	11,004 (75%)	9,211 (77%)	7,718 (76%)	8,849 (76%)	8,664 (78%)	5,755 (72%)	6,735 (74%)	7,316 (78%)	6,122 (60%)	6,311 (64%)	7,664 (66%)	7,550 (68%)	69,127 (80.4%)	-42,2
40-64 year olds(%)	4,066 (74%)	4,795 (72%)	5,149 (72%)	5,125 (74%)	6,609 (74%)	7,248 (79%)	5,233 (74%)	6,386 (76%)	7,579 (82%)	6,911 (72%)	6,656 (68%)	7,888 (71%)	7,675 (70%)	44,154 (85.6%)	88,8
65+ year olds(%)	659 (61%)	764 (64%)	1,225 (67%)	1,167 (64%)	1,499 (65%)	1,889 (71%)	1,425 (63%)	1,834 (65%)	2,530 (72%)	2,326 (59%)	2,557 (57%)	3,144 (55%)	3,401 (55%)	13,845 (68.3%)	416,1
Treatment measures															
All treatment measures	201,917	221,462	233,725	237,779	250,353	284,304	265,837	297,112	345,381	387,322	436,959	458,208	478,691	4,099,050	137,1
18-39 year olds(%)	132,597 (66%)	128,111 (58%)	120,310 (51%)	115,446 (49%)	115,012 (46%)	124,000 (44%)	109,858 (41%)	120,402 (41%)	131,943 (38%)	146,684 (38%)	161,760 (37%)	172,403 (38%)	183,847 (38%)	1,762,373 (43.0%)	38,7
40-64 year olds(%)	56,671 (28%)	79,040 (36%)	91,625 (39%)	98,086 (41%)	107,162 (43%)	124,392 (44%)	119,662 (45%)	134,113 (45%)	159,421 (46%)	178,897 (46%)	198,574 (45%)	203,350 (44%)	207,366 (43%)	1,758,359 (42.9%)	265,9
65+ year olds(%)	12,649 (6%)	14,311 (6%)	2,1790 (9%)	24,247 (10%)	28,179 (11%)	35,912 (13%)	36,317 (14%)	42,597 (14%)	54,017 (16%)	61,741 (16%)	76,625 (18%)	82,455 (18%)	87,478 (18%)	578,318 (14.1%)	591,6
Restorative treatment	56,817 (28%)	59,586 (27%)	60,288 (26%)	59,330 (25%)	60,727 (24%)	68,205 (24%)	65,715 (25%)	71,131 (24%)	79,672 (23%)	88,148 (23%)	101,838 (23%)	100,420 (22%)	96,895 (20%)	968,772 (23.6%)	70,5
Examinations	34,293 (17%)	33,781 (15%)	35,020 (15%)	34,930 (15%)	37,308 (15%)	42,906 (15%)	41,912 (16%)	48,476 (16%)	55,464 (16%)	64,488 (17%)	73,170 (17%)	76,494 (17%)	80,152 (17%)	658,394 (16.1%)	133,7
Radiology	20,466 (10%)	25,255 (11%)	26,908 (12%)	27,726 (12%)	33,392 (13%)	37,074 (13%)	31,604 (12%)	39,537 (13%)	46,451 (13%)	49,706 (13%)	59,414 (14%)	60,836 (13%)	71,506 (15%)	529,875 (12.9%)	249,4
Anaesthesia	25,533 (13%)	27,214 (12%)	28,183 (12%)	29,907 (13%)	30,279 (12%)	35,020 (12%)	34,361 (13%)	37,917 (13%)	43,133 (12%)	49,406 (13%)	56,739 (13%)	59,802 (13%)	63,675 (13%)	521,169 (12.7%)	149,4
Emergency treatment	9,655 (5%)	17,003 (8%)	22,804 (10%)	23,959(%)10	25,348 (10%)	27,199 (10%)	24,253 (9%)	25,197 (8%)	30,004 (9%)	33,534 (9%)	33,528 (8%)	37,268 (8%)	38,477 (8%)	348,229 (8.5%)	298,5
Periodontics	18,450 (9%)	18,902 (9%)	20,338 (9%)	19,953 (8%)	20,753 (8%)	23,141 (8%)	20,632 (8%)	20,463 (7%)	23,649 (7%)	29,404 (6%)	31,253 (7%)	35,554 (8%)	36,409 (8%)	318,901 (7.8%)	97,3
Endodontics	9,454 (5%)	9,620 (4%)	10,796 (5%)	12,191 (5%)	12,740 (5%)	14,758 (5%)	15,718 (6%)	17,596 (6%)	19,787 (6%)	22,218 (6%)	24,426 (6%)	23,957 (5%)	24,523 (5%)	217,784 (5.3%)	159,4
Oral surgery	7,730 (4%)	8,114 (4%)	8,929 (4%)	9,504 (4%)	9,152 (4%)	10,821 (4%)	10,721 (4%)	11,418 (4%)	14,130 (4%)	15,908 (4%)	17,544 (4%)	19,169 (4%)	21,427 (4%)	164,567 (4.0%)	177,2
Preventive care	11,720 (6%)	15,088 (7%)	13,697 (6%)	13,384 (6%)	13,315 (5%)	12,109 (4%)	10,208 (4%)	10,874 (4%)	10,047 (3%)	11,943 (3%)	13,362 (3%)	13,993 (3%)	11,143 (2%)	160,883 (3.9%)	-4,9
Other treatment	2,346 (1%)	2,233 (1%)	2,870 (1%)	3,214 (1%)	3,546 (1%)	8,666 (3%)	6,156 (2%)	9,457 (3%)	17,274 (5%)	16,207 (4%)	18,311 (4%)	22,647 (4%)	26,290 (5%)	139,217 (3.4%)	1020,6
Prosthetics	3,341 (2%)	2,771 (1%)	2,226(<1%)	1,995(<1%)	2,025(<1%)	2,350(<1%)	2,201(<1%)	2,395(<1%)	2,949(<1%)	3,384(<1%)	3,892(<1%)	4,121(<1%)	3,839(<1%)	37,489(<1%)	14,9
Treatment of TMJ disorders	1,083(<1%)	1,085(<1%)	1,140(<1%)	1,290(<1%)	1,431(<1%)	1,668(<1%)	1,910(<1%)	2,141(<1%)	2,201(<1%)	2,328(<1%)	2,781(<1%)	3,074(<1%)	3,320(<1%)	25,452(<1%)	206,6
Orthodontics	1,029(<1%)	810(<1%)	526(<1%)	396(<1%)	337(<1%)	387(<1%)	446(<1%)	510(<1%)	620(<1%)	648(<1%)	701(<1%)	873(<1%)	1035(<1%)	8,318(<1%)	0,6

During the study period, the proportion of those in need of basic caries and periodontal treatment (CPI > 2, D + d > 0) decreased slightly. A statistically significant decreasing trend could be found in the youngest age group (18–39 years) from 75 to 68% (*p* = 0.033*). In the age group 40–64 years the decrease was smaller, from 74 to 70% and the trend was not significant (*p* = 0.497). In the oldest age group (65 + years) the corresponding figures were from 61 to 55% (*p* = 0.394; Table 1; Table 3).

Altogether, 4,099,050 treatment measures were provided for the adults during the entire study period (Table 1). Almost equal shares were provided for the 18–39-year-olds (1,762,373, 43.0%) and 40–64-year-olds (1,758,359, 42.9%). The 65+ year-olds had had 578,318 (14.1%) treatment measures. The 18–39- year-olds had on average had 14,742, the 40–64- year-olds 23,015 and the 65+ year-olds 17,998 treatment measures per 1000 patients, respectively (Table 3).

Restorative treatment (968,772; 23.6%), examinations (658,394; 16.1%), radiology (529,875; 12.9%) anaesthesia (521,169; 12.7%) and emergency treatment (348,229; 8.5%) made up 73.8% of all treatment measures during the entire study period. Periodontal treatment (7.8%) and prevention (3.9%) made up smaller parts of the care provided and prosthetics, treatment of TMD disorders and orthodontics were extremely infrequent (fewer than 1%; Table 1).

As can be seen from Table 2, a major part of all preventive treatment (43.5%) was provided for the youngest adults and most periodontal treatment (45.5%), restorative treatment (45.8%) and prosthetics (52.0%) was for middle-aged adults. Other treatment categories were more evenly distributed among the age groups.

**Table 2:** **Table 2** Distribution (%) of treatment measures provided for adults in the five Finnish PDS units by patient age group (18–39 years, 40–64 years and 65+ years) for each of the main treatment domain during 2001–2013. Distribution (%) of treatment measures converted to treatment time using the Helsinki study on time used for different treatment measures [19] by age group

	Distribution of treatment measures by age, %				Distribution of treatment measures converted to treatment time by age, %			
Treatment measures	18-39 year olds	40-64 year olds	65+ year olds	Distribution of all treatment measures, %	18-39 year olds	40-64 year olds	65+ year olds	Distribution of all treatment measures converted to treatment time, %
Restorative treatment	39.1	45.8	15.1	23.6	38.8	46.4	14.8	28.4
Examinations	43.5	41.5	15.0	16.1	44.8	40.6	14.6	12.8
Radiology	44.8	43.4	11.8	12.9	45.1	42.7	12.2	3.2
Anaesthesia	52.4	38.3	9.3	12.7	52.6	38.2	9.2	4.2
Emergency treatment	41.1	44.0	13.9	8.5	41.0	44.8	14.2	9.2
Periodontics	39.0	45.5	15.5	7.8	36.2	47.6	16.2	15.2
Endodontics	45.4	44.8	9.8	5.3	46.1	45.0	8.9	11.6
Oral surgery	40.1	40.1	19.8	4.0	44.3	38.7	17.0	3.8
Preventive care	43.5	36.4	20.1	3.9	44.3	35.8	19.9	4.8
Other treatment	44.2	40.0	15.8	3.4	42.3	40.5	17.2	3.1
Prosthetics	11.3	52.0	36.7	0.9	9.2	55.2	35.5	2.7
Treatment of TMD disorders	43.9	46.2	9.9	0.6	46.9	45.3	7.8	0.9
Orthodontics	91.0	8.6	0.4	0.2	91.2	8.4	0.3	0.2
All	43.0	42.9	14.1	100	41.0	44.2	14.8	100

When treatment measures were converted into time [19], the share of periodontics doubled from 7.8 to 15.2% and endodontics from 5.3 to 11.6% respectively. The prosthetics share tripled from 0.9 to 2.7% but remained low. Radiology decreased from 12.9 to 3.2% and anaesthesia from 12.7 to 4.2%. The share of restorative treatment increased from 23.6 to 28.4% and preventive treatment from 3.9 to 4.8%. The share of examinations decreased slightly from 16.1 to 12.8% (Table 2). Treatments related to caries, restorative treatment (28.4%), examinations (12.8%), endodontics (11.6%) and emergency treatment (9.2%) made up 62.0% of dental personnel’s treatment time (Table 3).

**Table 3:** **Table 3** Numbers of treatment measures per 1000 patients provided for adults (> 17 years) combined by age group (18–39 years, 40–64 years and 65+ years) in the five PDS units from 2001 to 2013

	Year	2001	2002	2003	2004	2005	2006	2007	2008	2009	2010	2011	2012	2013
18-39y	All	5207	5537	5694	5820	5594	5642	5514	5972	5897	6372	7021	6697	6781
18-39y	RestorativeTreatment	1459	1451	1388	1355	1257	1216	1219	1263	1172	1230	1399	1224	1147
18-39y	Examinations	940	894	896	889	866	869	860	975	944	1076	1179	1103	1110
18-39y	Radiology	563	684	707	722	784	786	688	825	833	841	997	931	1079
18-39y	Anaesthesia	731	775	806	875	823	868	903	963	927	1023	1152	1095	1104
18-39y	EmergencyTreatment	213	383	525	569	539	524	486	501	498	525	530	546	536
18-39y	PreventiveCare	245	322	315	313	298	230	202	218	188	219	220	212	153
18-39y	Periodontology	482	463	454	440	415	405	371	332	339	441	427	459	447
18-39y	EndodonticTreatment	232	223	262	306	282	306	354	385	373	405	442	387	405
18-39y	TreatmentOfTemporomadibularDisorders	28	28	29	33	32	35	40	43	41	42	44	46	50
18-39y	Orthodontics	35	29	22	18	15	16	20	23	26	26	27	31	37
18-39y	Prosthetics	40	34	18	10	9	10	6	8	8	10	11	7	10
18-39y	OtherTreatment	63	64	82	89	91	182	153	211	319	290	318	371	406
18-39y	OralSurgery	175	185	191	200	183	194	211	224	228	243	274	284	297
40-64y	All	5806	6431	6519	6415	6105	6289	6172	6667	6721	7317	7926	7551	7495
40-64y	RestorativeTreatment	1668	1803	1773	1701	1573	1633	1646	1729	1676	1808	1976	1805	1648
40-64y	Examinations	871	892	909	888	865	929	970	1078	1064	1174	1299	1229	1227
40-64y	Radiology	553	698	719	731	817	812	743	901	908	961	1102	1028	1140
40-64y	Anaesthesia	597	656	668	683	631	665	692	747	754	850	947	919	941
40-64y	EmergencyTreatment	358	559	678	675	648	625	586	573	604	657	620	623	618
40-64y	PreventiveCare	416	487	391	363	292	214	169	183	139	177	205	177	130
40-64y	Periodontology	531	577	623	592	552	561	521	515	499	577	598	610	597
40-64y	EndodonticTreatment	315	324	323	345	331	339	371	397	392	431	448	418	389
40-64y	TreatmentOfTemporomadibularDisorders	33	33	33	36	39	38	49	52	45	45	57	54	55
40-64y	Orthodontics	13	11	5	2	1	1	2	2	1	2	2	2	1
40-64y	Prosthetics	159	107	81	70	62	66	69	63	70	74	83	79	64
40-64y	OtherTreatment	60	51	68	78	77	178	121	193	315	281	303	331	378
40-64y	OralSurgery	233	234	248	250	216	230	233	234	252	282	286	278	308
65+y	All	5872	5882	6443	5791	5316	5181	5174	5865	6015	6463	7284	6791	6701
65+y	RestorativeTreatment	1575	1577	1784	1541	1371	1323	1355	1497	1526	1636	1910	1673	1547
65+y	Examinations	865	877	977	887	815	785	849	984	1014	1153	1281	1236	1233
65+y	Radiology	336	347	554	534	553	541	495	657	698	718	841	756	821
65+y	Anaesthesia	505	499	522	504	427	403	420	477	501	530	616	565	592
65+y	EmergencyTreatment	340	522	644	559	547	480	458	491	505	564	550	531	525
65+y	PreventiveCare	659	678	455	387	387	408	414	386	283	270	301	312	262
65+y	Periodontology	468	449	590	519	478	455	449	471	471	538	612	601	594
65+y	EndodonticTreatment	219	196	213	201	215	192	210	254	238	247	286	225	213
65+y	TreatmentOfTemporomadibularDisorders	17	11	19	18	18	21	25	31	22	27	32	36	34
65+y	Orthodontics	4	2	0	0	0	0	0	0	0	0	1	0	0
65+y	Prosthetics	355	275	210	172	143	120	105	133	122	141	147	150	138
65+y	OtherTreatment	67	53	58	63	62	165	110	182	297	278	322	345	369
65+y	OralSurgery	463	395	418	407	300	288	285	302	338	360	387	361	373
All	All	5402	5849	6060	6048	5767	5839	5738	6250	6271	6793	7455	7069	7057
All	RestorativeTreatment	1520	1574	1563	1509	1399	1401	1418	1496	1447	1546	1737	1549	1428
All	Examinations	917	892	908	889	859	881	905	1020	1007	1131	1248	1180	1182
All	Radiology	548	667	698	705	769	761	682	832	843	872	1014	939	1054
All	Anaesthesia	683	719	731	761	697	719	742	798	783	866	968	923	939
All	EmergencyTreatment	258	449	591	609	584	559	523	530	545	588	572	575	567
All	PreventiveCare	314	398	355	340	307	249	220	229	182	209	228	216	164
All	Periodontology	494	499	527	508	478	475	445	430	429	516	533	549	537
All	EndodonticTreatment	253	254	280	310	293	303	339	370	359	390	417	370	362
All	TreatmentOfTemporomadibularDisorders	29	29	30	33	33	34	41	45	40	41	47	47	49
All	Orthodontics	28	21	14	10	8	8	10	11	11	11	12	13	15
All	Prosthetics	89	73	58	51	47	48	48	50	54	59	66	64	57
All	OtherTreatment	63	59	74	82	82	178	133	199	314	284	312	349	388
All	OralSurgery	207	214	232	242	211	222	231	240	257	279	299	296	316

v

**Fig. 1:**
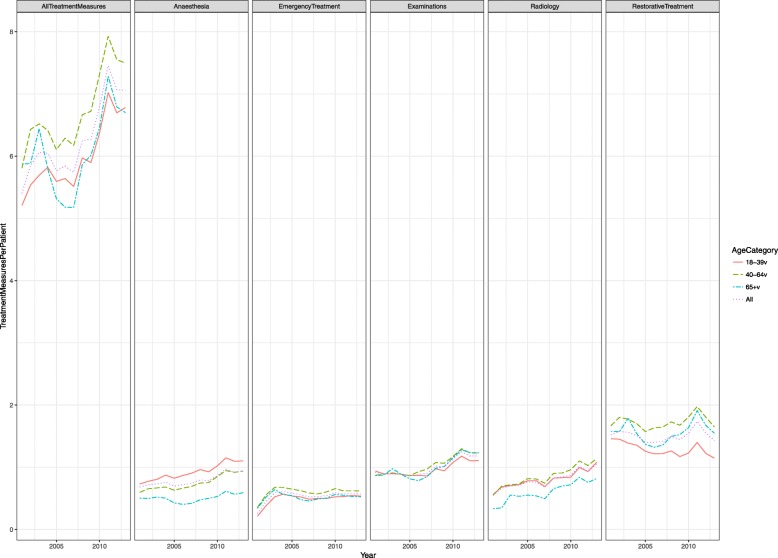
Numbers of total and the five most usual treatment measures (per patient) and all treatment measures (per patient) provided for adults (> 17 years) combined by age group (18–39 years, 40–64 years and 65+ years) in the five PDS units from 2001 to 2013

The total number of treatment measures provided increased from 5402 to 7057 per 1000 patients. Among the youngest age category (18–39 years), the mean number of treatment measures increased from 5207 to 6781 per 1000 patients, among the 40–64-year-olds from 5806 to 7495 per 1000 patients and among the oldest (65+ years) from 5872 to 6701 per 1000 patients (Fig. 1, Table 3).

Restorative treatment decreased from 1520 to 1428 treatment measures per 1000 patients, preventive care from 314 to 164 and prosthetics from 89 to 57 treatment measures.

Examinations increased from 917 to 1182 items per 1000 patients, radiology from 548 to 1054 items, anaesthesia from 683 to 939, periodontics from 494 to 537, emergency treatment from 258 to 567 and endodontics from 253 to 362 treatment measures respectively (Table 3).

Preventive treatment measures decreased among the 18–39-year-olds from 245 to 153 among the 40–64-year-olds; from 416 to 130 items and among the 65+ year-olds the decrease was from 659 to 262 treatment measures per 1000 patients per year.

A statistically significant increasing trend was found in the total number of treatment measures provided from 2001 to 2013 for the youngest age group (18–39 years) (*p* = 0.003**) and for the 40–64-year-olds (*p* = 0.015*). For the oldest group (65+ years), the trend was not statistically significant. The increasing trends in radiology (*p* < 0.001***), anaesthesia (*p* = 0.003**) and oral surgery (*p* = 0.004**) were statistically significant. The decreasing trend in preventive care was statistically significant (*p* = 0.003**; Table 4).

**Table 4:** **Table 4** Trend analysis on treatment need, on the number of treatment measures per patient in each treatment category provided for adult patients (> 17 years) and separately for the three age categories (18–39 years, 40–64 years and 65+ years) in the five PDS units. For the three separate age groups only statistically siategories are presented

Treatment need	Age category	mu	sd	t	p
In need of treatment	18-39v	-0.014	0.006	-2.442	0.033*
In need of treatment	40-64v	-0.004	0.005	-0.702	0.497
In need of treatment	65+v	-0.011	0.012	-0.887	0.394
Prevention vs. Treatment need					
No treatment need	All	0.078	0.035	2.197	0.050
No treatment need	18-39v	0.105	0.061	1.722	0.113
No treatment need	40-64v	-0.044	0.087	-0.505	0.623
No treatment need	65+v	0.147	0.208	0.707	0.494
In need of treatment	All	-0.046	0.122	-0.374	0.716
In need of treatment	18-39v	0.013	0.124	0.105	0.919
In need of treatment	40-64v	-0.063	0.107	-0.587	0.569
In need of treatment	65+v	-0.004	0.083	-0.053	0.958
All treatments and age categories					
All treatments	All the adults	0.022	0.007	3.114	0.009**
All treatments	18-39years	0.021	0.006	3.746	0.003**
All treatments	40-64years	0.021	0.007	2.864	0.015*
All treatments	65+years	0.011	0.018	0.618	0.549
Treatment categories and all ages					
RestorativeTreatment	All the adults	-0.003	0.009	-0.273	0.789
Examinations	All the adults	0.021	0.017	1.218	0.249
Radiology	All the adults	0.044	0.005	8.881	<0.001***
Anaesthesia	All the adults	0.027	0.007	3.671	0.003**
EmergencyTreatment	All the adults	0.066	0.051	1.279	0.227
Periodontology	All the adults	0.007	0.019	0.363	0.724
EndodonticTreatment	All the adults	0.030	0.021	1.390	0.192
OralSurgery	All the adults	0.035	0.010	3.571	0.004**
PreventiveCare	All the adults	-0.059	0.016	-3.711	0.003**
OtherTreatment	All the adults	0.176	0.014	12.374	<0.001***
Prosthetics	All the adults	-0.038	0.034	-1.130	0.282
Treatment of TMJ disorders	All the adults	0.048	0.006	8.046	<0.001***
Orthodontics	All the adults	-0.049	0.060	-0.815	0.432
For the three age categories, only statistically significant treatment categories are presented.					
Examinations	40-64years	0.030	0.010	3.083	0.010*
Radiology	18-39years	0.041	0.005	7.747	<0.001***
Radiology	40-64years	0.050	0.004	11.406	<0.001***
Radiology	65+years	0.070	0.012	5.561	<0.001***
Anaesthesia	18-39years	0.035	0.003	10.902	<0.001***
Anaesthesia	40-64years	0.038	0.009	4.174	0.002**
EndodonticTreatment	18-39years	0.051	0.011	4.761	<0.001***
OralSurgery	18-39years	0.044	0.011	4.055	0.002**
PreventiveCare	40-64years	-0.099	0.031	-3.155	0.009**
PreventiveCare	65+years	-0.074	0.019	-3.953	0.002**
OtherTreatment	18-39years	0.173	0.013	13.394	<0.001***
OtherTreatment	40-64years	0.183	0.016	11.602	<0.001***
OtherTreatment	65+years	0.183	0.024	7.768	<0.001***
Treatment of TMJ disorders	18-39years	0.049	0.005	10.281	<0.001***
Treatment of TMJ disorders	40-64years	0.048	0.007	6.685	<0.001***
Treatment of TMJ disorders	65+years	0.082	0.008	9.928	<0.001***

A statistically highly significant increasing trend was found in radiology for all age groups (*p* < 0.001***) through the years. When studying treatment profiles over patients’ age categories, there was an increasing trend in examinations provided for the 40–64-years-olds (*p* = 0.010*), in anaesthesia among the 18–39-year-olds (*p* < 0.001***) and the 40–64-year-olds (*p* = 0.002**). A statistically significantly increasing trend was found in endodontic treatment among the 18–39-year-olds (*p* < 0.001***) and in oral surgery among the 18–39-year-olds (*p* = 0.002**). The only treatment category having a statistically significantly decreasing trend was preventive care, among the 40–64-year-olds (*p* = 0.009**) and among the 65+ year-olds (*p* = 0.002**; Table 4).

There were on average almost five times (483.5%) more preventive treatment measures per patient among those not in need of treatment compared with those in need of treatment in every age group. In addition, among those in need of treatment there was a decreasing trend in preventive treatment measures per 1000 patients. Among the 40–64-year-olds from 2287 to 1383 (*p* = 0.569) and among the 65+ year-olds from 3759 to 1297 treatment measures per patient (*p* = 0.958; Table 3).

## Discussion

In Finland, many kinds of statistical information on the performance of the public dental services have been collected by the individual PDS-units. Recording of certain oral health indices considering treatment needs and treatment measures is mandatory and part of each PDS dentist’s salary is based on the treatment measures provided. Thus, data from the PDS records have been considered reliable [21]. There was little information about dental treatment provided in Finland before the national study in the year 2009 [11, 12]. The treatment profiles in the PDS units participating in this study were in line with the previously mentioned national study [11] indicating that the chosen units, covering 5.9% of the population, were not outliers among the Finnish PDS-units. The results of this study can thus be generalised to middle sized or big towns in southern Finland. A limitation is that no information on social background of the patients is collected in the PDS register and that the information on treatment needs and oral health indicators was rather crude.

The results showed that from 2001 to 2013, the number of adults (18+ years) treated in the participating PDS-units increased by 81.5%. In 2001, the shares of young (< 18 years) and adult patients were 51.5 and 48.5% respectively and in 2013 these were 36.8 and 63.2% [22]. This change was in line with the political intentions of the Dental Care Reform in 2001 aiming to improve adults’ access to the PDS.

Overall, during the 13-year study period, most adults living in the local municipalities (77.5%) had visited the PDS on some occasion. The legal obligation to organise emergency dental services for all inhabitants in its PDS uptake-area was included in the Dental Care Reform; this certainly explains a big part of the expanded use [23]. It was obvious from this study that most new patients were working age (18–64 years) adults. The share of older patients grew only from three to 12%. In 2000, 44% of the elderly were still edentulous in Finland [2]. During the study period, the number of dentists increased by 61.4% and the number of auxiliaries by 267.9% in the participating PDS units. The increased resources were used in treatment of adults only.

The study showed that the clinical treatment provided concentrated strongly on treating caries and its consequences. Examinations, restorative treatment, endodontics and emergencies made up 53.5% of all treatment measures and took 62.0% of the total treatment time of the staff during the whole study period. This can be regarded to be a disproportional share because the national epidemiological studies [1–3] have shown that, in addition to caries, gingivitis and periodontitis and great numbers of missing teeth even in anterior visible sectors without prosthetic devices are common in Finnish adults. Periodontal treatment made up only 7.8% of all treatment measures provided and 15.2% of the total treatment time. A worrying finding was that the share of preventive treatment was generally lower among those in need of treatment than those not in need of basic periodontal or restorative treatment.

The findings of the present study can be roughly compared with available data from the PDS in Sweden, where 46% of the 10 million treatment measures registered for the year 2017 in the PDS were examinations, 20% were periodontal, 16% restorative and 10% preventive treatment measures [24]. The corresponding values in this study in 2013 were 16.7, 7.6, 20.2 and 2.3%. It is evident that despite better oral health, Swedes received more examinations, periodontal and preventive treatment [2, 3, 5].

Also, in the private sector in Finland, restorative therapy dominates adult dental care although some more periodontal treatment is provided [12]. In general, the private sector is seen to provide more frequent and more comprehensive care to a smaller group of adult patients, whereas in the PDS more effort goes to examinations and emergency care and a greater proportion of adults receive irregular care due to long waiting lists and no recall system [12, 25].

Public dentists in Finland feel that their competence is weak in periodontal treatment [26]. This may be because dentists may think that much of this treatment should be given by the dental hygienists, but probably also because the PDS until 2002 catered mainly for children, adolescents and young adults. Lack of experience and skills is also likely to explain the fact that very little prosthetic treatment (0.2%) was provided by the participating PDS units. The cost of prosthetic treatment and especially fixed prosthetics has been high even in the PDS, because the technical work has to be bought from the private sector. The fact that some treatments are neglected also reflects lack of resources, especially specialists in adult dental care. Officially, prosthetics is included in the treatment palette of the PDS. Poor access to proper crown and bridge therapy and its high cost have resulted in restorative treatment practices where large composite fillings and crowns are used with wide indications, often leading to repetitive circle of restorative work and thus raising the share of restorative treatment [27]. Provision of questionable restorative treatment can also increase the need for endodontic treatment. In the national epidemiological study in 2000, 27% of the examined adults had at least one and 13% three or more teeth with apical periodontitis [2].

The public dentists (but not dental hygienists) have been encouraged to increase productivity by giving them salary increments from most treatment measures they have provided except radiography and preventive care. The salary increment is about 30–40% of the total wages. The most profitable treatments are and have long been examinations and restorative treatment. Thus, the findings reflect great discrepancy between the objectives of the Dental Care Reform in 2002 to give older adults born before the year 1956 access to the PDS and still continuing the use of old incentives aimed to steer productivity in treatment of young adults needing mostly treatment of caries when the incentives were created in 1980s.

Since this study period ended, a number of national best practice guidelines for most treatments in adult oral health care have been published to facilitate clinical treatment planning in Finland. These evidence-based recommendations include treatment of dental caries [28], temporomandibular disorders [29], restorative dentistry [30], dental infections [31] and prosthetics [32]. However, it is well-known that even the best guidelines will not become implemented automatically in daily practice but require education and leadership [33].

Overall, there has been little political pressure to look at the quality of adult dental care [34]. Chief medical officers, the superiors of the chief dental officers in the decentralised PDS organisation, are not sufficiently familiar with the challenges in adult dental care after the age restrictions were abolished in the PDS [35] and there has been no other interest group to drive this objective.

This study shows that use of routine administrative data collected from the databases of PDS organizations can improve transparency of oral health service delivery and give new tools for the managers and political leaders. The results also indicate that the PDS might be insufficiently resourced or the personnel is not efficiently used in providing care for adults. Besides the young, adults should also be included in a recall system in the PDS to guarantee improvement of their oral health. The present incentives connected with salary that favor selected treatment measures need to be replaced by a system that enables adequate comprehensive care and includes prevention.

## Conclusions

Adults’ dental treatment in the PDS concentrates on treatment of caries. Compared with the results of national epidemiological studies, periodontal treatment is insufficient and prosthetic treatment is almost totally neglected. The big increase in radiography suggests that the quality of examinations has improved. There was no significant decrease in treatment need except for the youngest adults. The unmet needs may be due to tradition, inadequate treatment processes, lack of resources or failed salary incentives.

## Data Availability

The data that support the findings of this study are available from the National Institute for Health and Welfare (THL) but restrictions apply to the availability of these data, which were used under licence for the current study, and are thus not publicly available. Data are, however, available from the authors upon reasonable request and with permission of the National Institute for Health and Welfare (THL) as well as the participating communities.

## Abbreviations

AR residual: Autoregressive residual
CPI: Community Periodontal Index
D: Number of permanent decayed teeth
DMFT: Number of decayed, missed and filled teeth
ns: Not significant
PDS: Public Dental Service
THL: The National Institute for Health and Welfare (Terveyden ja Hyvinvoinnin Laitos)
TMD: Temporomandibular disorders
